# Big data approaches to understanding gene regulatory networks in the shoot apical meristem and *de novo* shoot regeneration

**DOI:** 10.3389/fpls.2026.1837132

**Published:** 2026-05-28

**Authors:** Jack H. Carpenter, Emily L. Darby, Tamara Lechon, Simon Scofield

**Affiliations:** School of Biosciences, Cardiff University, Cardiff, United Kingdom

**Keywords:** bioinformatics, gene regulatory network (GRN), multiomics, organogenesis, regeneration, shoot apical meristem (SAM), plant development, transcriptomics

## Abstract

Gene regulatory networks (GRNs) regulate the development and function of the shoot apical meristem (SAM) in higher plants by controlling the division, differentiation, and developmental fate of meristematic cells. These GRNs are composed of transcription factors and their target genes, and are also coordinated by phytohormones, microRNAs, and epigenetic regulators. With the increasing integration of multiomic and single cell approaches, large amounts of data relating to SAM regulation have been collated, providing insight to the interactions between many GRN components. Whilst many well-established experimental approaches such as gene knockouts, transgene overexpression and reporter gene imaging still heavily contribute to the understanding of these networks, the use of large-scale datasets to construct GRNs has driven novel hypothesis generation and has been used to predict network topology and component interactions. Many of these components and interactions have subsequently been explored *in silico* and verified *in vivo*, emphasising the usefulness of data-driven approaches in studying the GRNs governing complex developmental processes. In this review, we highlight the interactions between some of the key SAM-associated transcription factors which act as network hubs both in normal plant development and in *de novo* shoot formation during regeneration, illustrating the importance of integrating multiple genome-wide approaches to generate robust GRNs and predictive models of SAM activity and *de novo* shoot regeneration.

## Introduction

Plant growth is a well-studied process that depends on the iterative production of new tissues and organs, such as leaves and flowers, throughout their lifespan. Organogenesis occurs from populations of stem cells in regions throughout the plant known as primary and secondary meristems, facilitating vertical and lateral growth, as well as recovery of growth following wounding during the process of regeneration. The shoot and root apical meristems (SAM and RAM, respectively) are primary meristems which house populations of pluripotent cells in highly organised stem cell niches. The SAM is situated at the shoot tip and is the control centre for multiple developmental processes, including stem cell maintenance, lateral organ initiation and meristem-organ boundary formation, which are controlled by complex gene regulatory networks (GRNs) that exhibit considerable crosstalk (Barton, 2010).

A GRN is a hierarchical set of interactions between different genes, proteins, and other molecules that control gene expression to govern a cellular process. The conceptualization of molecular interactions as GRNs has been fundamental to our understanding of plant systems (Espinosa-Soto et al., 2004; Vermeirssen et al., 2014; Taylor-Teeples et al., 2015; Wils and Kaufmann, 2017; Chávez-Hernández et al., 2022). They can be used to better understand how cell identity is determined and how perturbations to a system *in silico* may predict effects on a given biological process, driving novel hypothesis generation which can be investigated *in vivo*. In recent years, multiple studies have utilised computational techniques to analyse ‘big data’ produced by sequencing technologies to refine models of the SAM (Ikeuchi et al., 2018; Scofield et al., 2018; Ma et al., 2019; Zhang et al., 2021a; Chávez-Hernández et al., 2022; Sinha et al., 2023; Lechon et al., 2025) and have identified hub genes encoding key transcriptional regulators with high level connectivity within the GRN, such as the class-1 KNOTTED1-like homeobox (KNOX I), CUP-SHAPED COTYLEDON (CUC), WUSCHEL-like homeobox (WOX), PLETHORA (PLT) and TEOSINTE BRANCHED1-CYCLOIDEA-PCF1 (TCP) transcription factors.

GRNs are typically constructed using gene expression data and constitute a blueprint of plant regulatory systems, connecting nodes (representing genes) with edges (representing regulatory interactions). These blueprints can provide system-level insights into the stability and complexity of GRNs, including the measurement of topological features such as network size and node connectivity (see Van den Broeck et al., 2020 and Badia-i-Mompel et al., 2023 for reviews). GRN models typically use bulk gene expression profiling of multiple cell types within a given tissue or whole plant, although recent advancements in single cell technologies, as well as the combination of multi-omics data, such as chromatin landscaping data, has greatly improved GRN inference within the SAM (Ma et al., 2019; Satterlee et al., 2020; Zhang et al., 2021a).

The underlying regulatory systems of the SAM have been studied extensively utilising combinations of molecular genetics, modelling, and imaging techniques in the model organism *Arabidopsis thaliana* and many other plant species (Hata and Kyozuka, 2021; Kean-Galeno et al., 2024). Though work over the past 30 years has generated large amounts of transcriptomic data, helping to form a detailed understanding of the SAM, these GRNs remain incomplete. This is mostly attributable to the complexity of the interactions and the redundant functions of many components within GRNs that provide network robustness. This robustness is largely attributable to the considerable genetic redundancy within gene families of plants, which relates directly to the high level of gene duplication in many plant gene families (Pickett and Meeks-Wagner, 1995; Martienssen and Irish, 1999; Byrne et al., 2002; Geuten et al., 2011). Redundancy also extends to whole modules within GRNs, where entire gene circuits display parallel roles in development, correlating with phenotypic robustness (Alvarez-Buylla et al., 2010; Payne and Wagner, 2015; Challa et al., 2021). Furthermore, these networks function not only at the transcriptional level, but also at an epigenetic and proteomic level, depending on intracellular movement of proteins, transient protein interactions, phytohormone signalling, DNA methylation, histone modification and microRNAs (miRNAs) to control gene expression. Here we discuss the interplay between the different systems that regulate SAM function, highlighting some of the key regulatory hubs. We discuss how these hub genes are critical for both normal plant development and during the process of *de novo* shoot regeneration that occurs during *in vitro* plant tissue culture, which is used extensively for commercial plant micropropagation. Additionally, we outline the increasing dependence on computational techniques for the analysis of omics data to study the dynamic nature of SAM GRNs.

## SAM structure and functional domains

**Figure 1:**
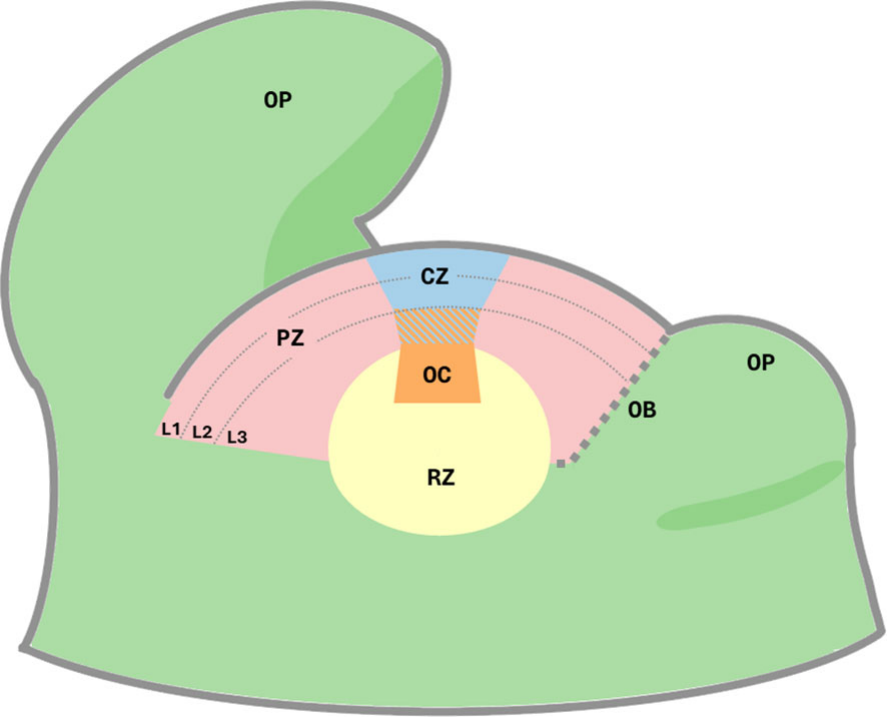
The structure of the shoot apical meristem (SAM). The origin of all apical growth, the SAM can be separated into zones that reflect functional roles. The pluripotent stem cells that give rise to new tissues are maintained in the central zone (CZ/blue). The number of stem cells in the CZ is regulated by the activities of the organising centre (OC/orange). The CZ and the OC overlap in the third cell layer (L3) of the SAM (blue and orange). Undifferentiated transit-amplifying cells in the peripheral zone (PZ/pink) undergo several rounds of division before committing to differentiation programmes and subsequently forming incipient organ primordia (iP) and ultimately, organ primordia (OP; green). The rib zone (RZ/yellow) gives rise to stem tissues. OB = organ boundary (grey dashed line).

The SAM is a three-dimensional, highly organised dome-like structure consisting of zones and layers that demarcate the different functions and fates of the constituent cells (**Figure 1**; Bowman and Eshed, 2000). Situated at the apex of the SAM, the central zone (CZ) contains the true stem cell population. Beneath the CZ is the organising centre (OC) which directs stem cell fate in the overlying stem cells in the CZ. Surrounding the CZ is the peripheral zone (PZ) of transit amplifying cells where organ founder cell specification and primordium initiation occur through accumulation of the phytohormone auxin and the activation of lateral organ-specific gene expression programmes (see Barton, 2010 for a review). The SAM is also organised into three clonally distinct layers superimposed on these zones, with the first and second layers (L1 and L2) undergoing anticlinal cell division and collectively forming the tunica tissue. The third layer (L3) undergoes a combination of anticlinal and periclinal divisions to comprise the corpus tissue. To prevent the incorporation of the CZ stem cells into new organ primordia, robust distinction between the cell populations in different zones is established and maintained to ensure proper development.

Multiple efforts have aimed to further define the functional domains of the SAM based solely on gene expression patterns (Heisler et al., 2005; Yadav et al., 2009, Yadav et al., 2014; Tian et al., 2019; Zhang et al., 2021a). Over the past 20 years, the methodology used has shifted from fluorescent reporter lines and spatial microarrays (Heisler et al., 2005; Yadav et al., 2009, Yadav et al., 2014), to whole genome and single cell approaches which have provided valuable community resources to explore SAM-specific expression data (Tian et al., 2019; Zhang et al., 2021a). Tian et al. (2019), used targeted purification of polysomal mRNA (TRAP-Seq) to extract ribosome-associated mRNAs, with tagged ribosomes expressed under putative domain-specific promoters, to elucidate the transcriptome of defined SAM domains (Tian et al., 2019). This study provides a valuable resource which can be used interactively to explore the translatome of domains within the SAM to drive novel hypothesis generation. However, the major drawback of these domain-level approaches is the use of bulk cell populations and marker genes to demarcate regions of the SAM. These methods often understate the complexity of cell populations, introduce biases (Zhang et al., 2021a) and also depend on a detailed prior knowledge of gene expression to select suitable promoters which is a challenge for under-studied species. Due to the resulting averaging of multiple cell types in bulk cell population studies, delineating cell fate trajectories of transit-amplifying cells with this type of data is not possible. Single-cell RNA-sequencing (scRNA-seq) of the SAM has provided a solution to this issue by resolving gene expression to the cellular level, transcending the need to define or group cell populations based on their locality within the SAM (Zhang et al., 2021a). Instead, cell populations are defined by similarities between individual transcriptome profiles. These data have been used to recapitulate multiple developmental trajectories of SAM tissues, to establish new regulators of shoot development and to map cell phylogenies from the zygote to adult plants using cell lineage tracing coupled with scRNA-seq (Xia et al., 2026). The data can also be explored interactively, making the data widely accessible to the research community (Zhang et al., 2021a). Additionally, the webserver ePlant facilitates the visualisation of AtGenExpress Consortium expression data for multiple stages of development (including the SAM) and CoNekT utilises coexpression data to aid in gene regulatory network visualisation (Waese et al., 2017; Proost and Mutwil, 2018).

## Regulatory modules in the SAM

**Figure 2:**
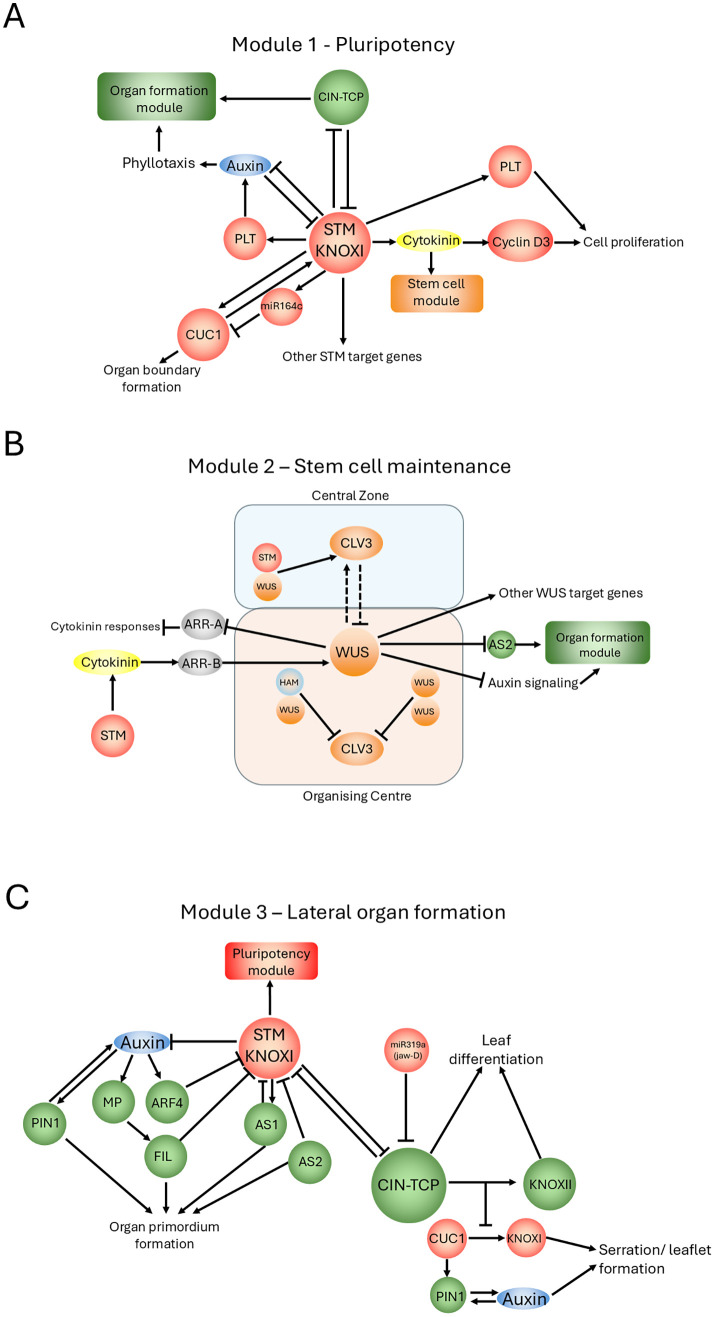
The shoot apical meristem gene regulatory network. **(A)** The pluripotency regulatory module (Module 1). KNOX I TFs (principally STM) repress the expression of the differentiation-promoting CIN-TCP TFs (part of the organ formation module; Module 3) in the SAM, thereby preventing cellular differentiation. STM promotes cytokinin biosynthesis which promotes cell proliferation via the CYCD3 pathway, and promotion of *WUS* expression in the stem cell maintenance module (Module 2). STM also promotes organ boundary formation via regulation of CUC1 and the CUC1-targeting microRNA, miR164C. STM directly regulates PLT7, which in association with other PLT factors, regulates cell division and the control of phyllotaxis via regulation of auxin levels. **(B)** The stem cell maintenance module (Module 2). *WUS* expression is induced by cytokinin and WUS acts to dampen cytokinin signalling via promotion of *ARR-A* expression. WUS activates *CLV3* expression in the CZ stem cells, either as a monomer or dimerised with STM. The ability of WUS to activate *CLV3* expression in the OC is reduced due to its homodimerisation or dimerisation with HAM. WUS represses auxin signalling and differentiation factors such as AS2 (part of the organ formation module) in the SAM. **(C)** The lateral organ formation module (Module 3). *STM* and other *KNOX I* genes are repressed by several factors, including CIN-TCPs, ARFs, FIL, AS1 and AS2, which promote organ primordium formation. CIN-TCPs act with KNOX II TFs to promote leaf differentiation and repress serration/leaflet formation via the CUC1-KNOX I module, which regulates auxin accumulation with PIN proteins. Factors in red are primarily involved with pluripotency, factors in orange are primarily involved in stem cell maintenance and factors in green are primarily involved in lateral organ formation. Positive regulation of gene expression is represented by arrow heads. Negative regulation is represented by flat heads.

The SAM is required to regulate multiple processes throughout shoot development, which are often explored in studies as isolated ‘regulatory modules’ such as those governing promotion of pluripotency, stem cell maintenance and lateral organ formation. In actuality, these systems depend on a considerable amount of interconnectivity and redundancy to ensure development proceeds properly (**Figure 2**). Moreover, several of the regulatory factors in these modules are also employed during shoot regeneration, leading to *de novo* formation of shoot meristems and lateral organs during *in vitro* tissue culture, and this is covered in later section of this review. Phytohormones play a critical role in controlling these regulatory developmental modules. In particular, auxin (indole-3-acetic acid) has a well-established role in promoting the initiation of lateral organ primordia in PZ cells on the flanks of the SAM (Reinhardt et al., 2003; Heisler et al., 2005; Jönsson et al., 2006), in addition to a critical role in mediating de-differentiation of cells during *in vitro* callus formation and root regeneration (Skoog and Miller, 1957). Another class of phytohormone, cytokinins, have a key role in the SAM and developing organ primordia where they promote cell proliferation and shoot formation during normal development (Riou-Khamlichi et al., 1999; Dewitte et al., 2007; Yang et al., 2021) and during *in vitro* shoot regeneration (Skoog and Miller, 1957). Both auxin and cytokinin exert their effect by mediating the transcriptional activation or repression of target genes associated with SAM or lateral organ development through well-characterised signalling pathways (reviewed in Yu et al., 2022a; Powell and Heyl, 2023).

### Module 1 - promotion of pluripotency in the SAM

A key property of the cells that comprise the SAM is the maintenance of pluripotency – the undifferentiated cell state where terminal cellular differentiation associated with the acquisition of specialised cell identities is blocked - thereby retaining the capacity to adopt a range of different cell fate trajectories. Pluripotency is a key property of the stem cells that reside in the CZ of the SAM, and also to a degree of the undifferentiated transit amplifying cells in the PZ before the specification of organ founder cell identity and the ensuing transition to differentiated cell types associated with lateral organ function (e.g. mesophyll cells, stomata, trichomes etc.). A key GRN for the promotion of pluripotency in the SAM, including stem cell specification, is coordinated by the class-1 *KNOTTED1-LIKE HOMEOBOX* (*KNOX I*) genes, which encode TALE-class homeodomain transcription factors (**Figure 2A**; Long et al., 1996; Belles-Boix et al., 2006). In *Arabidopsis thaliana*, class-1 (*KNOX I*) includes the four *KNOX* members *SHOOT MERISTEMLESS* (*STM*), *KNAT1* (also called *BREVIPEDICELLUS*; *BP*), *KNAT2*, and *KNAT6*. Class-2 *KNOX* genes (*KNOX II*) comprise *KNAT3*, *KNAT4*, *KNAT5* and *KNAT7*, and these are not involved in SAM development (Scofield and Murray, 2006) but instead have roles in leaf differentiation, root development and secondary cell wall biosynthesis (Reiser et al., 2000; Challa et al., 2021; Truernit and Haseloff, 2007; Nookaraju et al., 2022).

Multiple studies have provided greater insight into the functions of the *KNOX* classes through loss- and gain-of-function studies and the characterisation of expression domains, revealing that *KNOX I* genes share a significant amount of functional redundancy and display overlapping expression patterns (see Jia et al., 2023 for a review). In simple-leaved species such as Arabidopsis, *KNOX I* expression is generally restricted to, and is a key marker of, the SAM. *KNOX I* genes have a well-established role in the establishment and maintenance of the SAM, acting to promote pluripotency in stem cells and inhibiting cellular differentiation associated with lateral organ development in transit-amplifying cells (Long et al., 1996; Laufs et al., 2004; Belles-Boix et al., 2006; Guo et al., 2008; Spinelli et al., 2011; Scofield et al., 2014; Liu et al., 2018; Nidhi et al., 2021). Crucially, *KNOX I* gene expression is repressed in incipient organ primordia and developing organs, thereby allowing organ-specific differentiation programmes to occur. This transcriptional repression is mediated by multiple factors including the transcriptional repressor ASYMMETRIC LEAVES1 (AS1), the LOB-domain protein ASYMMETRIC LEAVES2 (AS2), members of the YABBY transcription factor family, the Polycomb Repressive Complex (PRC) and the class-2 TCP transcriptional regulators (Long et al., 1996; Ori et al., 2000; Byrne et al., 2002; Kumaran et al., 2002; Hay et al., 2006; Guo et al., 2008; Xu and Shen, 2008; Li et al., 2012; Yu et al., 2021).

*KNOX I* expression in Arabidopsis begins during the globular stage of embryogenesis, with the direct transactivation of *STM* by the NAC-domain transcription factors CUPSHAPED COTELYDONS (CUC1) and CUC2 which, in turn, are regulated by STM to establish the meristem-organ boundaries between cotyledons (see below; Aida et al., 1999; Spinelli et al., 2011; Aida et al., 2020). Additionally, STM directly regulates the paralogous *KNAT1/BP* and *KNAT2* genes, showing *KNOX I* members are capable of self-regulation at the level of the gene family. STM also acts to exclude expression of factors associated with organ differentiation and repression of *KNOX I* gene expression from the SAM, such as the class-2 *TCP* genes (Scofield et al., 2018). Initial *stm* knockout studies established the critical role of *STM* in stem cell identity and SAM formation which has been extensively corroborated over the last 30 years using a range of mutant alleles and RNAi-mediated approaches (Barton and Poethig, 1993; Endrizzi et al., 1996; Long et al., 1996; Aida et al., 1999; Lenhard et al., 2002, Lenhard et al., 2002; Cole et al., 2006; Spinelli et al., 2011; Landrein et al., 2015; Balkunde et al., 2017; Scofield et al., 2013; Su et al., 2020).

Numerous studies have sought to understand the role of *KNOX I* genes in coordinating SAM development using omics approaches to reveal directly regulated target genes and the topology of the GRN they comprise through analysis of KNOX-DNA interactions at target gene promoters. For example, in rice and maize the principal target genes of the KNOX I proteins OSH1 and KN1 were identified as being involved in phytohormone biosynthesis, signalling and response, especially relating to auxin and brassinosteroids (Bolduc et al., 2012; Tsuda et al., 2014; Yan et al., 2015).

In Arabidopsis and barley, *STM* or its ortholog *BKn3* have also been implicated in the regulation of auxin biosynthesis, transport and response, with several auxin-associated factors showing differential expression following ectopic expression (Richardson et al., 2016; Scofield et al., 2018). *KNOX I* genes have also been shown to mediate direct transcriptional repression of genes encoding gibberellic acid (GA) biosynthetic enzymes (Sakamoto et al., 2001; Hay et al., 2002; Chen et al., 2004; Jasinski et al., 2005; Song et al., 2021), thereby reducing levels of the phytohormone GA, which is detrimental to sustained SAM function. Additionally, STM has been shown to promote cytokinin biosynthesis in the SAM by activating expression of several members of the *ISOPENTYL TRANSFERASE* (*IPT*) gene family (Sakamoto et al., 2001; Chen et al., 2004; Jasinski et al., 2005; Yanai et al., 2005). This regulation of cytokinin biosynthesis is potentiated, in part, by the expression of the mitotic cell cycle enhancer *CYCD3;1* in response to cytokinin (Scofield et al., 2013). Hence, KNOX I proteins impinge on several phytohormone pathways that affect cell division, cell expansion and cell differentiation processes associated with SAM development/maintenance and lateral organ formation.

Using genome-wide chromatin immunoprecipitation (ChIP) analysis to identify directly-bound STM target genes, coupled with inducible up-regulation followed by transcriptomics analysis to identify STM-responsive genes, STM was shown to regulate the expression of many transcription factors associated with meristem development and the control of pluripotency (Scofield et al., 2018; Lechon et al., 2025). These included the class-1 *KNOX* genes *KNAT1/BP* and *KNAT2*, the AP2 family gene *PLT7/AIL7*, which promotes pluripotency and the regulation of phyllotactic patterning (Prasad et al., 2011; Mudunkothge and Krizek, 2012; Kareem et al., 2015), and *HB25* which promotes shoot identity (Hanano et al., 2020). STM was also shown to repress expression of the class-2 CINNCINNATA (CIN) subgroup TCP transcription factor-encoding genes *TCP3*, *TCP4* and *TCP10*, which promote cellular differentiation and themselves repress *KNOX I* gene expression during leaf development and antagonise meristem function when ectopically expressed (Koyama et al., 2007, Koyama et al., 2010; Li et al., 2012; Schommer et al., 2014; Li, 2025). This reveals a functional mechanism for the inhibition of cellular differentiation associated with leaf formation by KNOX I transcriptional regulators. Bayesian networking approaches were then used to depict the topological structure of the STM GRN and infer conditional dependencies among its constituent components, revealing potential regulatory relationships and interplay between transcription factors that drive SAM formation and function. For example, the network analysis using over 1000 publicly available transcriptomics datasets recapitulated established direct regulatory relationships between *STM* and *CUC1*, while also predicting novel regulatory relationships such as the regulation of *PLT7* and class-2 *TCP* expression by STM which have been verified experimentally (Spinelli et al., 2011; Scofield et al., 2018; Lechon et al., 2025).

### Module 2 - stem cell maintenance in the SAM central zone

One of the most extensively explored regulatory systems within the SAM is the WUSCHEL (WUS)/CLAVATA (CLV) pathway (Clark et al., 1996; Laux et al., 1996; Schoof et al., 2000; Yadav et al., 2011, Yadav et al., 2013; Zhou et al., 2015; Su et al., 2020). This regulatory module facilitates communication between the OC and the CZ to regulate the number of pluripotent stem cells within the central zone of the SAM (**Figure 2B**; Schoof et al., 2000; Brand et al., 2000).

*WUS* encodes a WOX-family homeodomain transcription factor that is critical for promoting stem cell identity in the SAM. Its expression is induced by cytokinin via the B-type ARABIDOPSIS RESPONSE REGULATORS (ARRs), and in turn it promotes cytokinin responses through transcriptional repression of A-type ARRs, which are repressors of cytokinin signalling (Laux et al., 1996; Ikeda et al., 2009; Leibfried et al., 2005; Meng et al., 2017). WUS is expressed in the OC where it migrates through the plasmodesmata to the cells in the L1 and L2 layers of the CZ, directly activating the transcription of *CLV3* (Yadav et al., 2011). *CLV3* encodes a small secreted peptide that in turn diffuses through the apoplast and binds to the CLV1 and CLV2-CORYNE (CRN) leucine-rich repeat receptor complexes, inducing a signalling cascade that represses *WUS* expression (Nikolaev et al., 2007; Bleckmann et al., 2010; Yadav et al., 2011; Hu et al., 2018). This negative feedback loop serves as a mechanism to maintain constant stem cell numbers and is subject to further layers of regulation. For example, it has been shown that at low levels, WUS monomers activate *CLV3* transcription by binding to a TAAT core in *cis*-regulatory elements (CREs) of *CLV3*, while at higher levels WUS forms homodimers which repress *CLV3* expression through the same CRE, and that differences in WUS protein stability contribute to this alternate regulation (Perales et al., 2016; Rodriguez et al., 2016; Sloan et al., 2020).

WUS has also been shown to interact with the GRAS domain-containing transcription factors encoded by the *HAIRY MERISTEMS1-4* (*HAM1-4*) genes, which are required for SAM function and regulate common target genes with WUS (Schulze et al., 2010; Zhou et al., 2015; Rodriguez et al., 2016, Rodriguez et al., 2024). *HAM* expression overlaps with *WUS* in the OC of the SAM but is not detected in the epidermal cells in the CZ (Zhou et al., 2018; Gruel et al., 2018). While WUS monomers function to activate *CLV3* expression in the CZ stem cells, the WUS-HAM heterodimers that form in the OC are crucial for repressing *CLV3* expression in this region (Zhou et al., 2015, Zhou et al., 2018; Rodriguez et al., 2016) but have not been shown to directly bind *CLV3 cis*-regulatory elements. Instead, this interaction is important for controlling the activity, stability and diffusability of WUS (Rodriguez et al., 2024), which likely affects its ability to modulate target gene expression. Further insight into the stem cell homeostatic mechanism controlled by WUS, CLV3 and HAM was achieved with the use of computational and ordinary differential equation (ODE) models (Gruel et al., 2018; Zhou et al., 2018). The ODE model describing *WUS*, *CLV3*, and *HAM* expression patterns in the SAM successfully recapitulated the biological system, also replicating shifts in expression domains introduced by perturbations seen in knockout backgrounds or in the context of axillary meristem formation (Gruel et al., 2018). The “pocket repressor” model predicted WUS monomer activation of *CLV3* in the CZ but WUS-HAM dimer repression of *CLV3* in the OC, where WUS and HAM are both expressed, and that WUS-HAM interactions are sufficient to modulate the WUS-CLV3 negative feedback loop to ensure stem cell homeostasis and patterning of the stem cell niche (Gruel et al., 2018). Further studies have described the roles of additional components in controlling *WUS* and *CLV3* expression. For example, members of the ERECTA family of receptors and EPIDERMAL PATTERNING FACTOR-LIKE ligands have been shown to constrain the expression domains of *WUS* and *CLV3* within the SAM, and mathematical modelling has been used to study these regulatory interactions to produce refined models of stem cell regulation (Kimura et al., 2018; Liu et al., 2020; Zhang et al., 2021b; Uzair et al., 2024; Shpak and Uzair, 2025).

A recent study used a multiomics approach to elucidate the mechanisms that generate conducive chromatin landscapes for the function of the WUS-CLV system in floral meristems (FM) (Hawar et al., 2025). With cleavage under targets and tagmentation (CUT&Tag) and ChIP-seq, it was determined that the histone acetyltransferase (HAT) GENERAL CONTROL NON-DEREPRESSIBLE5 (GCN5) is required for the addition of active H3K9Ac marks onto *WUS* and *CLV3* loci chromatin (Stockinger et al., 2001; Poulios and Vlachonasios, 2018; Hawar et al., 2025). The necessity of GCN5 in the activation of the WUS-CLV system in FMs was further consolidated in the study with the use of RNA-seq on *gcn5–7* mutants, compared with the wild type. *gcn5–7* tissue saw a reduction in the expression of *WUS* and *CLV3* as well as many other flower development-related genes (Hawar et al., 2025). It would, therefore, be interesting to see the similarities in which the WUS-CLV-HAM-promoting chromatin landscape is established in the SAM compared to the FM. Multiomics has been further employed to investigate the mechanism by which WUS functions as a transcriptional regulator, combining ChIP-seq and RNA-seq timecourse experiments with genome-wide DNase hypersensitive sites to identify and validate direct transcriptional targets (Ma et al., 2019). These approaches revealed that WUS acts on target loci through regulation of histone acetylation, providing insight into the functional mechanisms of this complex GRN (Ma et al., 2019). Future exploration of this WUS-CLV-HAM network could benefit from the incorporation of additional predictive approaches such as Bayesian network analysis using the abundant transcriptome and epigenome data to investigate network logic as well as novel components of the GRN.

The converging meristem maintenance roles of STM and WUS, as well as overlapping expression domains, has long alluded to interaction between these two homeodomain transcription factors (Brand et al., 2002; Gallois et al., 2002; Scofield et al., 2014; Su et al., 2020). Furthermore, though *WUS* expression precedes *STM* during embryogenesis, STM is required to sustain *WUS* expression in the SAM, potentially through promotion of cytokinin biosynthesis, as *WUS* expressing cells in the OC are recruited into organ primordia upon downregulation or loss of *STM* expression (Scofield et al., 2014). Furthermore, controlled perturbations to *WUS* expression led to an equivalent response in *STM* expression, implying *STM* is regulated to some extent by WUS (Su et al., 2020). Recent studies have explored the intersection between STM and WUS GRNs, showing that STM also directly regulates *CLV3* expression by binding a TGACA motif in the *CLV3* promoter, in close proximity to the WUS-regulated TAAT motif (Su et al., 2020) and downstream of the *CLV3* gene body (Lechon et al., 2025). Su et al. (2020) showed that STM dimerization with WUS is required to bind the *CLV3* promoter and activate its transcription. Furthermore, Su et al. (2020) suggest that due to the similar contribution of STM and WUS at *CLV3 cis*-regulatory regions, but different requirements during different growth stages, the WUS-STM regulation of *CLV3* acts dynamically throughout development. The overlapping presence of STM and WUS in the OC, and the absence of *CLV3* expression, is thought to be a result of WUS-HAM dimers preventing WUS-STM dimer formation (Su et al., 2020). Incorporation of STM into the pocket-repressor model in different developmental contexts could enhance understanding of SAM formation and maintenance. Other omics approaches such as single cell assay for transposase-accessible chromatin (ATAC-seq) and RNA-seq data could be combined to provide a cellular-level context to this GRN by revealing differences in chromatin accessibility and gene expression between SAM domains (Dorrity et al., 2021; Bang et al., 2025).

### Module 3 - lateral organ formation

As rigid plant cell walls prevent cell mobility, organogenesis depends on lateral displacement of meristem cells towards the PZ following division, where distinct groups of transit amplifying cells undergo cell-type specification to form new lateral organ primordia that give rise to new leaves or flowers. Lateral organ primordia are initiated iteratively from the SAM in a predictable pattern termed phyllotaxis, a process which is heavily dependent on the localised accumulation of the phytohormone auxin (indole-3-actetic acid). The generation of auxin maxima on the flanks of the SAM has been studied extensively with the use of mathematical models (Jönsson et al., 2006; Smith et al., 2006; de Reuille et al., 2006; Pernisová and Vernoux, 2021). Importantly, PIN-FORMED (PIN) proteins facilitate polar auxin transport and form a positive feedback loop with auxin to direct PIN1 proteins to cells displaying a high auxin response (Reinhardt et al., 2003; Jönsson et al., 2006). Coincident with this, stable repression of the expression of pluripotency genes at sites of incipient organ primordia is required for the activation of organ-associated differentiation programmes. Hence, the formation of an auxin maximum coincides with the repression of *STM* expression and the activation of transcription factors associated with organ specification and differentiation, such as AS1(**Figure 2C**; Byrne et al., 2002; Hay et al., 2006).

The precise geometrical positioning of incipient primordia is commonly observed as the 137.5° angle between successive organs in Arabidopsis, giving rise to spiral phyllotaxis. This iterative process is regulated, in part, by members of a AP2/ERF transcription factor family subclade comprising *AINTEGUMENTA* (*ANT*), *AINTEGUMENTA-LIKE6*/*PLETHORA3* (*AIL6*/*PLT3)*, *AIL5/PLT5*, and *AIL7*/*PLT7* (Prasad et al., 2011; Mudunkothge and Krizek, 2012). In *ant-4 ail6–2 ail7–1* triple mutants, the SAM initiates a few leaves before termination whereas *plt3 plt5 plt7* triple mutants fail to establish spiral phyllotaxis, instead adopting a metastable state with lateral organs initiating in two alternating rows (Prasad et al., 2011; Mudunkothge and Krizek, 2012). These PLT transcription factors exhibit overlapping expression domains in the SAM and have been shown to regulate auxin maxima from within the OC, independently from *PIN1* regulation (Pinon et al., 2013). Instead, PLT transcription factors control *YUCCA* (*YUC*)*1* and *YUC4* genes encoding flavonoid monooxygenases which catalyse a rate limiting step in auxin biosynthesis (Won et al., 2011; Pinon et al., 2013). Furthermore, it has been shown through ChIP-seq and GRN analysis that STM is a direct regulator of *PLT7*, linking stem cell maintenance and phyllotaxis (Scofield et al., 2018; Lechon et al., 2025).

The auxin response factors (ARFs) ETTIN, and ARF4 have been shown to directly repress *STM* and *KNAT1* expression via histone deacetylation during the induction of flower primordium in the SAM during reproductive growth, whereas the ARF MONOPTEROS (MP) represses *STM* and *KNAT1* indirectly through *FILAMENTOUS FLOWER* (*FIL*) (Chung et al., 2019). These class B ARFs recruit histone deacetylases that displace acetyl groups from H3 tails, leading to chromatin compaction and *STM* silencing (Chung et al., 2019). Early *in situ* hybridisation assays depicted the expression boundaries of leaf identity-related genes such as *FIL* and *YABBY3* (*YAB3*) which appear mutually exclusive to the expression of *STM* (Eshed et al., 2004; Nole-Wilson and Krizek, 2006) though more recent scRNA-seq analysis has suggested that a subpopulation of transit-amplifying cells with a cell fate trajectory towards leaf identity express both *STM* and leaf identity-related genes (Zhang et al., 2021a), suggesting that their interaction is more complex and that ARFs might necessitate specific chromatin landscapes to silence *STM*.

Through a combination of ChIP-seq and RNA-seq, it was shown WUS represses auxin accumulation in SAM stem cells to low levels through de-acetylation of histone H3K9/K14 residues (Ma et al., 2019). This de-acetylation coincided with WUS binding sites in the 5’ UTRs and transcriptional start sites of many auxin response genes such as multiple *INDOLE-3-ACETIC ACID INDUCIBLE* genes as well as *ARF4*, allowing WUS to regulate the size of the SAM (Ma et al., 2019). This WUS-mediated repression of auxin is de-repressed by MP during floral organogenesis (Wu et al., 2015; Ma et al., 2019). Interestingly, the CLV proteins also play a less-established role in the regulation of organ primordia formation via interactions with auxin response pathways. Under certain environmental conditions, some *clv1* null mutant alleles display a primary inflorescence termination phenotype, suggesting that CLV1 has an additional role in promoting auxin-dependent meristem maintenance (John et al., 2023). The application of RNA-seq to *clv3* mutants showed a subtle change in auxin-responsive genes suggesting that CLV peptides promote a low auxin regime in the meristem to promote primordia outgrowth, similar to the *PLT* genes (Won et al., 2011; Pinon et al., 2013; Chung et al., 2019; John et al., 2023). These studies emphasise the importance of maintaining a low level of auxin in the meristem for proper maintenance of the SAM and how auxin maxima are required for the repression of *STM* and *WUS* to activate organ formation programmes. The repression of pluripotency factors such as *WUS* and *STM* provides space for pluripotency antagonists to exert changes to chromatin states that induce organ primordia identity.

Examples of pluripotency antagonists are *BLADE-ON-PETIOLE1/2* (*BOP1* and *BOP2*) genes (Ha et al., 2007) and the *AS1* and *AS2* genes (Byrne et al., 2002; Guo et al., 2008; Machida et al., 2022). *BOP1* and *BOP2* encode proteins with BTB/POZ domain and ankyrin repeats, and directly induce expression of the LOB domain-encoding transcription factor *AS2* at the leaf base to ensure correct development of leaf morphogenesis (Ha et al., 2007). Interestingly, *STM* overexpression has been shown to increase *BOP1/2* expression, further validated by the binding of STM to the *BOP* promoter regions, suggesting a negative feedback loop between STM and BOP (Scofield et al., 2018; Lechon et al., 2025).

*AS1* encodes a MYB-domain transcriptional repressor that acts as a competitive regulator to, and directly bound target of, STM and WUS (Ma et al., 2019; Lechon et al., 2025). During organogenesis, AS1 works in complex with AS2 to repress *KNAT1/BP* and *KNAT2* expression, thereby excluding *KNOX I* expression from developing organ primordia (Yadav et al., 2013). AS1-AS2 heterodimer complexes directly bind to two non-redundant regions of these *KNOX I* promoters and recruit HOMOLOG OF HISTONE CHAPERONE (HIRA), a chromatin remodelling factor to induce a repressive chromatin state which further inhibits enhancer activity during organogenesis (Guo et al., 2008). A recent study has shown that, despite the mutually antagonistic functions and mutually exclusive expression patterns of *STM* and *AS1*, STM binds to the *AS1* promoter region and promotes its expression, albeit at a relatively moderate level (Lechon et al., 2025). This highlights the complexity of some of the regulatory relationships among pluripotency- and differentiation-promoting genes, suggesting the involvement of complex feedback regulation to build stability into regulatory sub-modules within the SAM.

Through yeast two-hybrid and ChIP experiments, AS2 was shown to facilitate the binding of TCP transcription factors, which repress *KNOX I* and *CUC* gene expression and act to promote differentiation (Li et al., 2012). TCPs are often generalised as heterochronic regulators of leaf development with a large amount of functional redundancy within the gene family. This diverse family transforms environmental input to internal signalling which facilitates the plasticity of multiple developmental programmes (Li et al., 2012). Of the 24 members of the TCP family in *Arabidopsis thaliana*, 5 TCPs in the *CIN* subclade are post transcriptionally regulated by the microRNA miR319 (Palatnik et al., 2003). The dominant *miR319* mutant (the so-called *jaw-D* mutant), in which levels of *miR319a* are higher than normal, develops leaves with highly serrated and curled margins, similar to *KNOX I* overexpression phenotypes (Palatnik et al., 2003, Palatnik et al., 2007; Schommer et al., 2014; Koyama et al., 2017). The functional redundancy of these so-called *CIN-TCPs* was revealed through mild to no observable phenotypes in single knockouts studies, corroborated by *jaw-D* mutant analysis which affects a greater number of *TCP* genes at once, further illustrating their importance in leaf development (Bresso et al., 2018). Multiple studies have focused on TCP3 and TCP4 as a models to further elucidate the role the miRNA-regulated *CIN-TCP*s in leaf primordia, particularly in the regulation of transcriptional targets (Koyama et al., 2007; Efroni et al., 2008; Koyama et al., 2010; Sarvepalli and Nath, 2011; Li et al., 2012; Efroni et al., 2013; Li and Zachgo, 2013; Challa et al., 2016; Koyama et al., 2017; Vadde et al., 2018; Dong et al., 2019; Challa et al., 2021; Shankar et al., 2023).

The JAW-targeted TCPs play a substantial role in the formation of leaf margin serrations in simple leaf species and leaflet formation in compound leaf species (Crawford et al., 2004; Hu et al., 2023), and do so via the regulation of *CUC* and *KNOX I* genes (Laufs et al., 2004; Hay and Tsiantis, 2006; Koyama et al., 2007, Koyama et al., 2010; Wang et al., 2022). The mechanism in which CUC genes regulate margin development through *PIN1* has been examined in detail by combining RNA-seq, ChIP-seq, and DNA affinity purification (DAP-seq) with mathematical modelling. Through this it was demonstrated that, between species, the variation of *CUC1* genes and their endogenous promoters are sufficient to give rise to differences in leaflet complexity (Nikovics et al., 2006; Koyama et al., 2007, Koyama et al., 2010; Bilsborough et al., 2011; Hu et al., 2024). This JAW-TCP-CUC regulatory module ensures simple leaf development by repressing the KNOX I-CUC-auxin system (Challa et al., 2021). In parallel to this module are the redundant roles of the evolutionarily distinct *KNOX II* family members. Expression of *KNOX II* genes is observed in leaves but not the SAM, with *knat3,4,5* mutants displaying deeply serrated leaves (Challa et al., 2021). Furthermore, simultaneous repression of *JAW-TCP*s and *KNOX II* members leads to reactivation of the STM-CUC module and initiates supernumerary leaflet emergence in leaves, similar to compound leaf development, further establishing the role of CIN-TCPs in the suppression of meristem identity factors (Challa et al., 2021).

More recently, CUC1 has been linked, through *in vivo* studies and computational models, to the coordination of auxin maxima in the SAM and leaves during organ development (Hu et al., 2024; Kong et al., 2024). In the floral meristem, CUC1 modulates the speed of PIN1 repolarisation, increasing the intensity of the auxin maxima it surrounds and focuses PIN-dependent auxin maxima to promote rapid sepal initiation (Kong et al., 2024). Comparatively, during leaflet development, CUC1 and CUC2 provide a similar input to orchestrate auxin-regulated margin morphology such as leaf serrations in *Arabidopsis thaliana* and leaflets in *Cardamine hirsuta* (Bilsborough et al., 2011; Hu et al., 2024; Kong et al., 2024). Together, these studies show the importance of KNOX I-regulated localisation of *CUC* expression to the organ boundary at the SAM periphery, as well as PIN1 polarisation by CUC1 to generate sites for the initiation of new organ primordia.

The proper generation of lateral organs is also highly dependent on transcription factor coordination to generate meristem-organ boundaries to separate opposing GRNs delineating meristem cells from those in organ primordia. One family of organ boundary transcription factors comprises the *CUC* gene family (*CUC1*, *CUC2*, and *CUC3* genes), which are initially required for the activation of *STM* to establish the embryonic SAM (Takada et al., 2001; Aida et al., 1999). STM and CUC1 have been shown to constitute a positive feedback loop that specifies organ boundary cell identity between the SAM and lateral organ primordium (Spinelli et al., 2011; Balkunde et al., 2017; Scofield et al., 2018). Despite the predominant expression of *STM* throughout the central and peripheral zones of the SAM, and the expression of *CUC1* being primarily localised to the organ boundary, STM and CUC1 have been shown to bind to each other’s respective promoter sequence and activate transcription. A study involving live SAM imaging and mathematical modelling showed that this is potentially attributable to STM also indirectly activating expression of the CUC1-targeting microRNA, *miR164c* (Scofield et al., 2018). This generates an incoherent feed-forward loop where STM both activates CUC1 directly, and indirectly activates its repressor. The different read-outs of gene expression of this regulatory system in the SAM (STM) and organ boundary (CUC1) are attributable to the requirement for STM mobility to create an instructive gradient across the meristem, with high levels in the CZ and a gradual decline towards the incipient organ primordium. *CUC1* expression is much more sensitive to STM levels than *miR164c*, and so even at very low levels, such as those adjacent to the organ primordium, STM can activate *CUC1* expression. *miR164c*, however, is not as sensitive to STM levels, so only tends to be activated where STM levels are relatively high, such as throughout the majority of SAM cells. As such, miR164c levels are low adjacent to organ primordia, but not in the rest of the SAM, and so *CUC1* is observed to be expressed only in these boundary cells.

Non-mobile STM variants display *CUC1* expression spread throughout the SAM as well as organ fusion defects (Balkunde et al., 2017). The dependence of STM mobility has also been independently corroborated though simulation of *STM*, *miR164c*, and *CUC* mRNA and protein expression dynamics in the SAM with an ordinary differential equation (ODE) model (Scofield et al., 2018). STM mobility has been linked to the binding of FT INTERACTING PROTEIN (FTIP) 3 and FTIP4 to control endosomal trafficking of STM to the plasma membrane, therefore mediating intracellular and intercellular movement of STM (Liu et al., 2018). Loss of FTIP3 and FTIP4 results in STM being trafficked to the plasma membrane and reduces intercellular recycling back to the nuclei of shoot cells (Liu et al., 2018). This model illustrates that the informative STM gradient requires STM mobility and autoregulation to achieve the proper localisation of CUC1 to the meristem-organ boundary (Balkunde et al., 2017; Scofield et al., 2018). Once an organ boundary is established, *STM* expression is activated in the organ boundary cells and quantitatively correlates with increasing tissue folding, highlighting the role of mechanical forces in boundary formation (Landrein et al., 2015). *STM* expression at the organ boundary is induced by mechanical stress and is partially decoupled from auxin signalling, further highlighting the complexity of the interaction between auxin and STM.

## *De novo* shoot regeneration

The GRNs described above control the fate of cells in the SAM, a process that is canonically dependent upon the existence and maintenance of the population of stem cells housed within the SAM which possess a high differentiation potential. Plants also have the capacity to undergo cell fate reprogramming, whereby cells which are already differentiated can dedifferentiate, and subsequently undergo *de novo* shoot regeneration (Perianez-Rodriguez et al., 2014; Ikeuchi et al., 2016).

**Figure 3:**
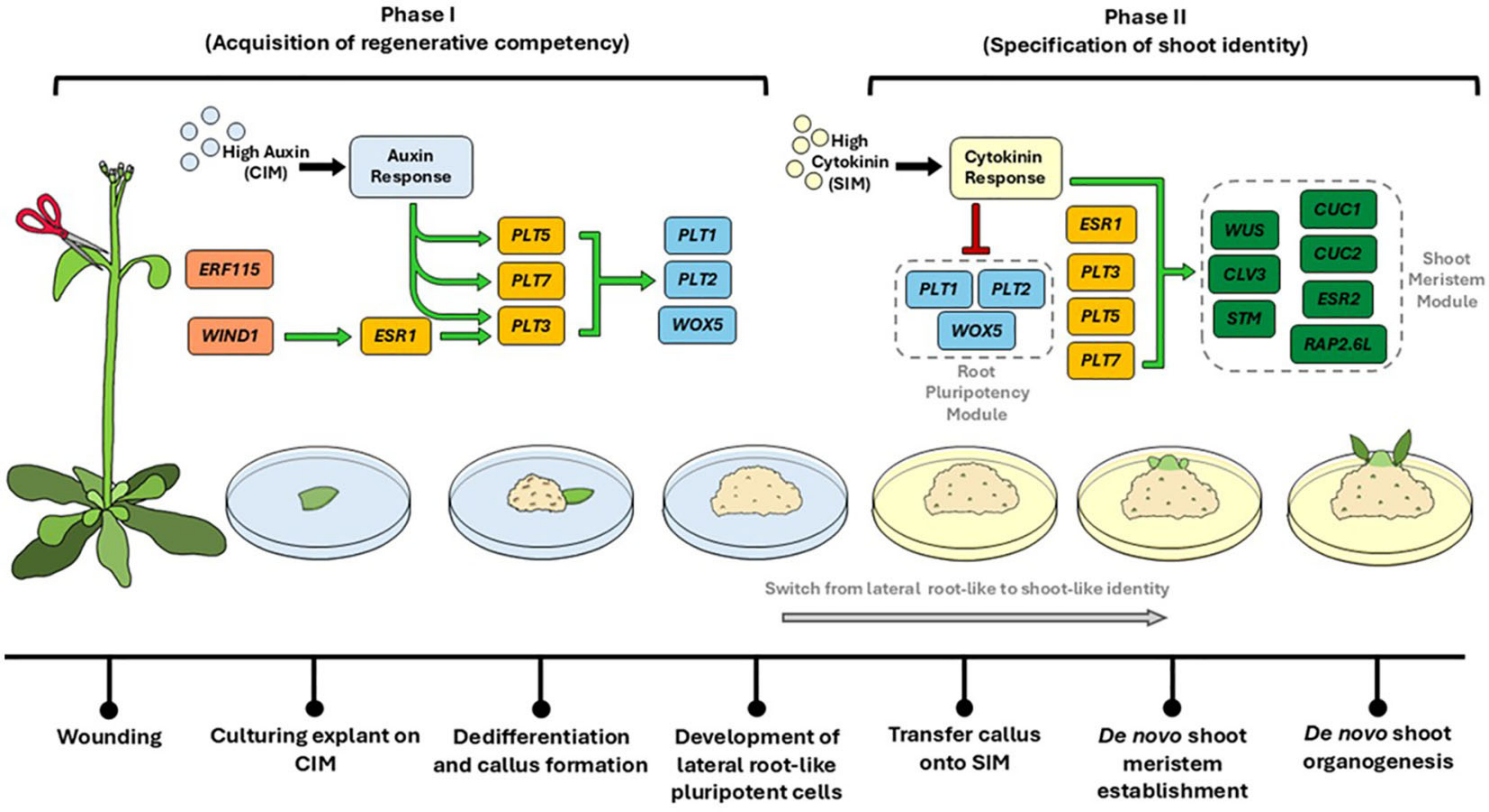
Roadmap of *in vitro de novo* shoot regeneration in *Arabidopsis thaliana*. A sequential overview of *de novo* shoot organogenesis is illustrated from left to right, beginning with wounding and transfer of a leaf explant onto auxin-rich callus induction medium (CIM). Wound sites initiate local callus formation, within which lateral root-like pluripotent cells develop. Upon transfer to cytokinin-rich shoot induction medium (SIM), a key transcriptional switch from lateral root-like to shoot-like identity is triggered, driving the establishment of *a de novo* shoot meristem and subsequent shoot organogenesis. The roadmap is divided into the two principal phases of regeneration: Acquisition of regenerative competence (callus formation and dedifferentiation), and specification of shoot identity (meristem establishment and shoot development). Transitions between these phases are visually marked, with transcriptional modules and hormonal cues collaboratively guiding cellular reprogramming and fate specification throughout regeneration. Key phytohormone signalling cues and transcription factor activities are depicted above each stage: wound-inducible transcription factors are highlighted in orange; transcription factors involved in acquisition of regenerative competence are marked in gold; root pluripotency-related transcription factors are marked in blue; shoot apical meristem and shoot-identity-related transcription factors are highlighted in green. Auxin and cytokinin cues are also marked in light blue and light yellow respectively.

*De novo* shoot regeneration takes place as part of a wound-response pathway, driving regeneration of damaged tissue to ensure survival. *De novo* shoot regeneration occurs in two key phases associated with different gene expression programmes (**Figure 3**). Typically, a wounding event triggers an initial cascade of wound-response genes to be expressed, causing cells at the site of wounding to dedifferentiate and reacquire pluripotency, as well as increase cell proliferation (Ikeuchi et al., 2017; Rymen et al., 2019). The secondary phase then involves the expression of shoot-related genes, which drive the acquisition of shoot identity amongst the dedifferentiated mass of cells, eventually establishing a *de novo* shoot meristem from which replacement shoot organs can arise (Tian et al., 2018).

Since *de novo* regeneration in plants is heavily dependent upon an interplay between modulation of endogenous phytohormone homeostasis and the activity of multiple transcriptional programmes, plant regeneration can also be achieved *in vitro.* Explants can be cultured on sequential phytohormone-rich media, which in turn can induce expression of genes following a similar transcriptional programme to wound-responsive regeneration signalling (Skoog and Miller, 1957; Ikeuchi et al., 2017). Explants are initially cultured on auxin-rich callus induction media (CIM), which instigates a signalling cascade, causing the cells to dedifferentiate to form a callus. The callus is then transferred onto cytokinin-rich media, called ‘shoot induction media’ (SIM), which results in the upregulation of shoot identity genes (**Figure 3**; Cary et al., 2002; Atta et al., 2009; Wu et al., 2022).

### Genetic control of shoot regeneration

There are many genes which have been implicated at various stages of *de novo* shoot regeneration, most of which have key roles in normal SAM development and function, indicating that plant regeneration must require tightly regulated crosstalk between TFs belonging to a variety of different transcriptional programmes and networks, including phytohormone signalling, wound and stress response, stem cell maintenance and organogenesis. Initial wounding triggers the expression of a number of *WOUND INDUCED DEDIFFERENETIATION (WIND)* genes, namely *WIND1*, which upregulates *ENHANCER OF SHOOT REGENERATION 1 (ESR1)*, both of which are essential in driving cellular reprogramming during shoot regeneration through the reacquisition of pluripotency (Banno et al., 2001; Iwase et al., 2011, Iwase et al., 2017). The *PLETHORA* genes, *PLT3, PLT5* and *PLT7*, whose role in promoting pluripotency and controlling phyllotactic pattering was described earlier, have also been identified as key regulators across both stages of *de novo* shoot regeneration. During the initial stage, *PLT3,-5* and -*7* upregulate *PLT1* and *PLT2* to establish regenerative competency, which relates to the canonical role of *PLT1* and -*2* in establishing pluripotency in root stem cells (Kareem et al., 2015). Furthermore, the first phase of *de novo* shoot regeneration follows a similar transcriptional trajectory to that of lateral root primordia development, including expression of *WOX5* which drives pluripotency acquisition (Sarkar et al., 2007; Pi et al., 2015). At a precise stage of this lateral root-like development, cytokinin signalling can initiate a transcriptional switch, which in turn suppresses the lateral root development transcriptional programme. Cytokinin signalling then acts to prime the cells to commit to shoot cell fate, therefore the precise timing of this cytokinin-driven switch acts as an essential determinant of the shoot regenerative capacity of these cells (Che et al., 2006; Sugimoto et al., 2010; Rosspopoff et al., 2017; Kim et al., 2018). This type of developmental stage-dependent cell fate reprogramming has also been identified during axillary meristem initiation (Wang et al., 2017).

Once the cells at the site of *de novo* shoot regeneration have acquired regenerative competence, cytokinin signalling along with expression of *ESR1*, *PLT3, PLT5* and *PLT7* act to upregulate a series of canonically shoot-related genes, including *WUS, STM, CLV3, CUC1* and *CUC2*, as well as *RAP2.6L* and *ESR2* (Wu et al., 2022). The increase in expression of these key shoot-related genes in turn results in suppression of the previously expressed root-related genes which are involved in initial pluripotency acquisition (Banno et al., 2001; Kareem et al., 2015). *WUS* has long been proposed to be a master regulator of *de novo* shoot organogenesis, as ectopic *WUS* expression has been found to be sufficient to induce *de novo* shoot organogenesis in roots (Gallois et al., 2004; Zhang et al., 2017). Overlapping roles between *ESR1, PLT3* and *PLT5* in regulating *CUC1* expression during *de novo* shoot organogenesis have also been suggested, and while previous studies have established that *ESR1* does not act upstream of the *PLT* genes, the potential relationship between these genes has yet to be investigated (Iwase et al., 2017).

### Shoot regeneration GRNs in Arabidopsis

It is clear that there are several overlapping transcriptional programmes which play crucial roles in regulating the two phases of *de novo* shoot organogenesis, and therefore there are likely to be multiple GRNs which interact to precisely regulate this process to ensure successful shoot regeneration (**Figure 3**). Moreover, shoot regeneration employs a reconfiguration of the GRNs involved in shoot development described earlier in the review. In recent years, research into plant regeneration has been forced to take a broader approach to begin to encapsulate the many different potential TFs involved in modulating this process (Ikeuchi et al., 2018; Lardon et al., 2020; Bae et al., 2022; Wu et al., 2022).

The first major plant cellular reprogramming GRN was constructed by Ikeuchi et al. (2018), using enhanced Yeast One-Hybrid (eY1H) assays to establish a more integrated view of the variety of transcription factors which underpin the multiple pathways involved in reprogramming. Relationships between 252 TFs and 48 promoters were identified within the GRN, from which three TFs were labelled as key ‘nodes’ which underpinned the majority of the GRN interactions. These key nodes defining the network were PLT3, ESR1 and HEAT SHOCK FACTOR B1 (HSFB1). The GRN constructed from the eY1H assays suggested that these genes responded to common upstream signalling to orchestrate downstream developmental decisions, with PLT3 and ESR1 forming a robust and overlapping network with multiple shared downstream targets. Furthermore, the network determined that while ESR1 and PLT3 act in distinct yet overlapping networks, ESR1 may also act upstream of PLT3, which contrasts with previous observations (Ikeuchi et al., 2017). Multiple other AP2/ERF family members were also interlaced throughout the proposed GRN, including some *AP2/ERF* genes which had not previously been characterised with respect to plant regeneration and cellular reprogramming. This finding indicated that further functional studies should investigate the roles of these other transcription factors within the AP2/ERF family, as novel regulators of wound-induced reprogramming could be identified (Ikeuchi et al., 2018).

In 2020, a Genome-Wide Association Study (GWAS) was performed using 190 different *Arabidopsis thaliana* accessions to establish which key genes best described variation in regenerative capacity between the accessions (Lardon et al., 2020). Five master regulators of regeneration were identified within the study, each of which presented allelic differences which were likely to be associated with the variation in *de novo* regeneration across the accessions studied (Lardon et al., 2020). The most important regulator was *WUS*, supporting the previous studies naming *WUS* as a master regulator of *de novo* SAM formation (Gallois et al., 2004; Gordon et al., 2007; Chatfield et al., 2013; Wang et al., 2017; Zhang et al., 2017). Single nucleotide polymorphism (SNP) variation within the *WUS* promoter sequence were found to correlate with the efficiency of shoot regeneration by altering the number of ARR binding motifs, increasing potential *WUS* expression in response to cytokinin signalling. The second most important regulator of regeneration in the study was *AT3G09925*, a Pollen Ole e 1 (POE1) allergen and extensin family gene. Despite having never been previously implicated in plant regeneration, a strong correlation between SNPs within this gene and variation in regenerative capacity suggested that this could present as a key gene which needs to be considered in future research to understand *de novo* shoot regeneration. Other master regulators identified by Lardon et al. (2020) included *LSH4*, *CLE2* and *WAVY GROWTH E3 LIGASE* genes.

A transcriptional regulatory dynamics analysis was performed by Wu et al. (2022), combining multiple approaches, including regeneration *cis-*element analysis, chromatin state and expression profiling, and developmental genetics to elucidate TFs involved in *in vitro* shoot regeneration. While motif enrichment analysis confirmed activity of TFs involved in auxin signalling in explants on CIM, and activity of cytokinin signalling TFs and WUS on SIM, some novel positive regulators of *de novo* shoot regeneration were also identified. *Cis*-element analysis paired with stage-specific chromatin accessibility peaks suggested that IDD/JACKDAW (JKD) is a positive regulator of *de novo* shoot regeneration. MYC, PIF, MYB93 and NGA2 were also identified as essential positive regulators of *de novo* shoot regeneration. This was supported by further functional studies of mutants of these genes, which resulted in defective shoot regeneration (Wu et al., 2022).

### Shoot regeneration GRNs in crop species

Similar transcriptomic and multiomic studies have also been conducted in other plant species, including wheat and tomato, identifying which genes are likely to be central regulators of *de novo* shoot regeneration based upon their conservation between different plant species (Larriba et al., 2022; Liu et al., 2023). A GRN for *de novo* organ regeneration from young tomato hypocotyl explants was constructed using RNA-seq data to investigate transcriptional differences between the basal and apical regions of the explants (Larriba et al., 2022). Multiple homologous genes could be identified between the GRNs for tomato and *Arabidopsis* regeneration, including *WUS, WIND1, ESR1* and *ESR2*, along with orthologs for *PLT3, PLT7* and *WOX5*. This indicates that the underlying GRN which control *de novo* regeneration in plants is shared beyond the Brassicaceae family.

Despite sharing a common core GRN, a few transcription factors with novel roles in *de novo* regeneration were identified in tomato, which have not previously been associated with regeneration in *Arabidopsis.* CDF3, which is normally involved in drought and osmotic stress response in *Arabidopsis*, was identified as a key TF involved in *de novo* shoot regeneration in the tomato explants. While the function of CDF3 in tomato *de novo* regeneration was unclear, Larriba et al. (2022) suggested that CDF3 may play a role in a metabolic switch to alter phytohormone homeostasis, facilitating the cell fate reprogramming. While CDF3 has not been reported in Arabidopsis regeneration, the potential role in tomato *de novo* regeneration still highlights the shared crosstalk between phytohormone signalling, and stress and wounding responses, which orchestrate reprogramming in plants.

A more extensive study into *in vitro* wheat regeneration was conducted, using a multiomics approach to generate GRNs from RNA-seq, ATAC-seq and CUT&Tag-seq datasets (Liu et al., 2023). A general conservation of genes which are involved in early regeneration were identified, further confirming the conservation of key regulators within the GRN between different plant species. Interestingly, SNPs occurring within a WOX family gene were found to be associated with different rates of callus dedifferentiation between different wheat varieties. This supports the evidence described by Lardon et al. (2020), highlighting the impact of SNPs within a few key regulators on the overall regenerative capacity of different varieties of the same plant species. Despite the general conservation of genes involved in callus induction between wheat and Arabidopsis, the study did find some disparities during the early stage of callus formation.

During early callus formation in *Arabidopsis*, LBD and NAC family genes which are involved in early auxin signalling were upregulated, meanwhile in wheat, DOF and G2-like genes were upregulated at this stage. Further analysis into the role of DOF genes identified that specific DOF genes could increase regenerative efficiency in wheat, revealing potential novel targets for improved wheat regeneration. Liu et al. (2023) then went on to investigate whether any of these genes could present as candidate novel positive regulators of wheat regeneration, identifying *TaDOF5.6* and *TaDOF3.4* as two genes which should be investigated in future studies looking to improve regeneration in other wheat varieties.

### Resolving overlapping functions in regeneration and development.

A limitation of these studies investigating *de novo* regeneration, which was highlighted by Ikeuchi et al. (2018), is that many of the key regulatory genes which have been identified within these proposed regeneration GRNs also play key roles in other biological and developmental processes in plants. *PLT3* and *ESR1*, among many other genes which have been described as key components of these GRNs, are also involved in normal organogenesis in plants. It is therefore an important consideration that some of the studies into TF motif binding could also present binding to normal organogenesis targets along with *de novo* regeneration targets. Ikeuchi et al. (2018) therefore called for investigation into how physiological conditions could be altered to uncouple the different roles of these TFs to specifically investigate their roles in the context of *de novo* regeneration (Ikeuchi et al., 2018).

In recent years, a web-based application called ‘REGENOMICS’ has been designed for analysis of plant regeneration-related GRNs, comprising a multitude of different tissue-specific and developmental stage-specific transcriptomic datasets, which specifically relate to plant regeneration. These include bulk RNA-seq datasets as well single-cell RNA-seq (scRNA-seq) datasets, primarily for *Arabidopsis thaliana*, however the database has expanded to begin to include datasets from other plant species too. This application enables users to explore single and multi-query analyses of co-expression networks, and GRNs, which should assist in future endeavours to unravel the multilayered GRNs which interact to modulate plant regeneration (Bae et al., 2022).

Wu et al. (2022) also emphasised the importance of single-cell studies, such as scRNA-seq datasets to investigate the precise transcriptional trajectory of cells within a pluripotent population. This would remove the distortion in bulk RNA-seq datasets which arises due to the lack of homogeneity across the cell populations in calli, since only a small subpopulation of cells within a callus actually undergo full cell fate reprogramming (Wu et al., 2022). Such studies could be used to compare and determine whether a true transcriptional association exists between wound-induced and phytohormone-induced regeneration. Dataset collections such as REGENOMICs with its expanding collection of datasets from non-*Arabidopsis* plant species could also be used to investigate conservation of key regeneration regulators. This could be used to identify orthologs for key regulators in recalcitrant plant species, which may elucidate novel targets to attempt to regenerate tissue from commercially important plant species that are sterile or difficult to propagate using traditional vegetative cloning methods.

## Future perspectives

The field of developmental biology aims to understand the highly organised and complex processes that govern cellular differentiation and tissue formation. In plants, these processes employ GRNs to maintain stem cells to provide a constant supply of new cells for the formation of shoot and root tissues. Thanks to a combination of genetic, molecular, and imaging approaches substantial insight into the formation and development of the SAM have now been achieved (**Figure 2**). Additionally, networking approaches and mathematical models have frequently been used to probe our understanding of apical systems *in silico*. Since the ‘Omics’ revolution, a significant contribution to these discoveries is a result of the generation of ‘big data’ produced by advances in high-throughput sequencing techniques and decreasing sequencing costs. GRNs employing machine learning or Bayesian algorithms are now making use of the large volumes of transcriptomic data, and are ever-improving with the integration epigenetic data (such as ChIP data), increasing precision and accuracy by honing in on direct, causal interactions (Chen et al., 2020; Klein et al., 2020; Sauta et al., 2020; De Clercq et al., 2021; Lechon et al., 2025).

On a similar note, an investigation into regulators of reactive oxygen species (ROS) signalling used a supervised machine learning method to incorporate seven GRNs comprised of transcription factor motif, open chromatin, co-expression, and ChIP-binding data to create an integrative GRN (iGRN) (De Clercq et al., 2021). The iGRN also incorporated conserved non-coding regions across 13 dicotyledonous genomes using a comparative motif mapping algorithm (Van de Velde et al., 2016). The iGRN was able to recapitulate known regulatory interactions as well as predicting novel ones, later validated *in vivo*. As this model outperformed the individual input models, it highlights how using a broad range of omics data can vastly improve network inference. It will be interesting to see how the use of such networks will shed more light on conserved network hubs between plant species and core facets of shoot development.

Despite the widespread use of these advanced techniques to investigate SAM processes, features of experimental design such as the use of bulk tissue and single timepoint “snapshot” studies have limited their potential. As a result, a true representation of the continuous and dynamic nature of SAM GRNs as well as the cross-talk between different systems remains elusive (Zhang et al., 2021a). Trends in experimental design are now rapidly shifting towards the integration of multiple genome-wide approaches with single cell analysis to analyse stem cell differentiation in the SAM (Scofield et al., 2018; Sijacic et al., 2018; Ma et al., 2019; Tian et al., 2019; Mahajan and Yadav, 2020; Zhang et al., 2021a) and in developing floral meristems (reviewed in Pelayo and Yamaguchi, 2023). Furthermore, insights into the genome-wide chromatin landscape of the SAM is now being explored at the bulk tissue and single cell level (Zhang et al., 2012; Zhu et al., 2015; Sijacic et al., 2018). Future single cell studies of SAM chromatin and organ primordia tissues will provide greater insight to the chromatin landscape. Combining this with other epigenetic and transcriptomic data would contribute to resolving these GRNs to the cellular level.

Due to the nature of high-throughput sequencing data, vast amounts of transcriptome and chromatin accessibility information have been generated and uploaded to public repositories for community use. For example, the Plant Public RNA-seq Database compiles ~100,000 RNA-seq libraries across multiple plant species to explore gene co-expression across multiple tissues and experimental conditions (Yu et al., 2022b). Likewise, the Arabidopsis Shoot Cell Atlas hosts the published scRNA-seq data from Zhang et al. (2021), providing an interactive interface with the data. SAM research will greatly benefit from leveraging these large single cell omics datasets with the latest bioinformatics tools, though current GRN techniques will likely require new innovations to supplement the spatial data produced by scRNA-seq (Zinati et al., 2024).
